# Molecular tools for differentiation of non-typeable *Haemophilus influenzae* from *Haemophilus haemolyticus*

**DOI:** 10.3389/fmicb.2014.00664

**Published:** 2014-12-02

**Authors:** Janessa Pickering, Peter C. Richmond, Lea-Ann S. Kirkham

**Affiliations:** ^1^School of Paediatrics and Child Health, The University of Western AustraliaPerth, WA, Australia; ^2^Centre for Vaccine and Infectious Disease Research, Telethon Kids Institute, The University of Western AustraliaPerth, WA, Australia

**Keywords:** *Haemophilus haemolyticus*, NTHi, identification, culture, molecular differentiation

## Abstract

Non-typeable *Haemophilus influenzae* (NTHi) and *Haemophilus haemolyticus* are closely related bacteria that reside in the upper respiratory tract. NTHi is associated with respiratory tract infections that frequently result in antibiotic prescription whilst *H. haemolyticus* is rarely associated with disease. NTHi and *H. haemolyticus* can be indistinguishable by traditional culture methods and molecular differentiation has proven difficult. This current review chronologically summarizes the molecular approaches that have been developed for differentiation of NTHi from *H. haemolyticus*, highlighting the advantages and disadvantages of each target and/or technique. We also provide suggestions for the development of new tools that would be suitable for clinical and research laboratories.

## Introduction

Identification and taxonomic classification of *Haemophilus* species can be challenging (Norskov-Lauritsen, 2014). This is particularly true for *Haemophilus haemolyticus*, which is often misidentified as non-typeable *H. influenzae* (NTHi) despite significant differences in pathogenicity. In a landmark study in 2007, Murphy et al. identified NTHi isolates with altered culture phenotypes from patients with chronic obstructive pulmonary disease (COPD) (Murphy et al., 2007). To test the hypothesis that the phenotypically different NTHi isolates were also genetically different, the authors analyzed 490 culture-defined NTHi isolates. Using a combination of genetic and immunological techniques, they found that the variant isolates were actually non-hemolytic *H. haemolyticus*, a closely related respiratory tract commensal that appears similar to NTHi via culture. The NTHi misidentification rate was significant, with 27% (12/44) of nasopharyngeal isolates and 40% (102/258) of sputum isolates misidentified as NTHi by culture. Analysis of 130 culture-defined laboratory NTHi isolates from middle ear effusion found that none were *H. haemolyticus*. Further analysis of 58 invasive isolates from the United States national collection identified 4 *H. haemolyticus* isolates that were previously characterized as a cryptic genospecies. This study reaffirmed *H. haemolyticus* as a respiratory tract commensal that is rarely cultured from sterile sites and highlighted the issue of NTHi misidentification by culture to the scientific community. Since 2007, retrospective analysis of phenotypic NTHi isolates from other studies have identified similar rates of misidentification (Chang et al., 2010; Kirkham et al., 2010; Hare et al., 2012; Pickering et al., 2014b).

The impact of NTHi misidentification is far-reaching given that NTHi-positive cultures are used to diagnose chronic suppurative otitis media and exacerbations of COPD, prescribe antibiotics, and estimate the efficacy of treatments and preventative strategies for NTHi disease including vaccines. Furthermore, the misidentification of *H. haemolyticus* as NTHi has potentially impacted on estimations of the proportion of antibiotic resistant NTHi (Witherden et al., 2013).

X (hemin) and/or V (β-nicotinamide adenine dinucleotide) factor requirement is routinely used in diagnostic laboratories to distinguish between *Haemophilus* species, however NTHi and *H. haemolyticus* require both X and V factors. The principal phenotypic difference between NTHi and *H. haemolyticus* is the production of a hemolysin by *H. haemolyticus* allowing species differentiation on blood agar plates (Kilian, 1976b). However, this difference is often unreliable as *H. haemolyticus* can lose the defining hemolytic phenotype upon passage (Sandstedt et al., 2008), or the hemolytic phenotype may be absent from the outset. Earlier, it was suggested that *H. haemolyticus* is a hemolytic variant of *H. influenzae* (Broom and Sneath, 1981). However, modern phylogenetic studies have identified clear species differences (McCrea et al., 2008; Norskov-Lauritsen, 2011). It is now widely accepted that culture alone cannot reliably distinguish NTHi from *H. haemolyticus.*

An ideal molecular tool for NTHi and *H. haemolyticus* differentiation is one that is rapid, robust, inexpensive, requiring standard laboratory equipment, and limited technical expertise. A superior tool would be one that unambiguously determines whether an isolate is NTHi or *H. haemolyticus* in a single reaction to reduce the time and cost for identification. However, the development of such tools for NTHi and *H. haemolyticus* differentiation has been difficult due to extensive genetic similarities between these species. Over the last decade, considerable research effort has focused on identifying molecular targets and suitable methodologies to differentiate NTHi from *H. haemolyticus*. A chronological review of each potential target and discussion of the advantages and disadvantages of the methodologies is given below and summarized in Table 1.

**Table 1:** **Summary of gene targets and methodologies used to discriminate *H. influenzae* from *H. haemolyticus***.

**Target**	**Method**	**Number of isolates**		**References**	**Outcomes**
		***H. haemolyticus***	***H. influenzae***		
*16SrDNA*	DNA seq, PCR	114 (102 SP, 12 NP)	318 (156 SP, 32 NP, 130 MEE)	Murphy et al., 2007	Dendrogram of *16SrDNA* sequences clusters NTHi and *H. haemolyticus* separately. *16SrDNA* PCR yields unequivocal results for ~90%
	DNA seq	7 (1 ref, 5 SP, 1 dental plaque)	8 (1 PS, 1 SP, 1 UN, 1 CSF, 3 blood, 1 LS)	Norskov-Lauritsen et al., 2009	*H. influenzae* strains cluster but there is no clear resolution for *H. haemolyticus* and other closely related strains
	PCR	1 (LS)	12 (LS)	Abdeldaim et al., 2009	*16SrDNA* PCR amplified *H. influenzae* and not *H. haemolyticus*
	DNA seq	7 (1 ref, 5 SP, 1 dental plaque)	8 (1 PS, 1 SP, 1 UN, 1 CSF, 3 blood, 1 LS)	Norskov-Lauritsen, 2011	Increased level of *16SrDNA* gene polymorphism in *H. haemolyticus* and other non-NTHi strains makes classification with *16SrDNA* difficult
	DNA seq	44 (44 RT)	78 (78 RT)	Theodore et al., 2012	Six distinct phylogenetic groups observed^b^
	PCR, DNA seq	28 (27 NP, 1 reference)	27 (21 NP, 6 refs)	Binks et al., 2012	*16SrDNA* PCR results in equivocal isolates. NTHi and *H. haemolyticus 16SrDNA* genes cluster separately with equivocal strains falling in between both species
			11 equivocal (NP)		
	seq of concatenated *16SrDNA* and *recA* genes	20 (PS-asymptomatic)	52 (5 PS-asymptomatic, 47 PS-URTi)	Zhu et al., 2013	*16SrDNA*-*recA* phylogeny is used to compare new differentiation methods of NTHi/*H. haemolyticus* such as MALDI-TOF (which is much faster)
	FISH	7 (1 ref, 2 SP, 1 BAL, 1 PS, 1 U, 1 GF)	50 (8 SP, 9 NP, 8 TS, 4 BAL, 4 PS, 6 ES, 5 NS, 1 BS, 2 EARS, 1 MSS, 1 ref)	Frickmann et al., 2013	*16SrDNA* gene probes specific for NTHi and *H. haemolyticus* resulted in 85% correct identification
*sodC*	PCR, Southern blot, Dot blot, IEF	20^a^	20^a^	Fung et al., 2006	*sodC* only found in *H. haemolyticus* and can be used for differentiation
	Hybridization blots	1 (LS)	480 (241 RT, 161 MEE, 68 CS, 10 from other sites-2 blood)	Norskov-Lauritsen, 2009	1.6% of *H. influenzae* isolates encoded *sodC*
	Microarray, MLSA, IEF, Southern blot, DNA seq	110^a^	169^a^	McCrea et al., 2010a	*sodC* found in 9% of NTHi strains and should not be used to discriminate NTHi and *H. haemolyticus*
*ompP6*	DNA seq, Immuno-blot, 7F3 MAb	6^*c*^	12^*c*^	Murphy et al., 2007	*H. haemolyticus* strains lack 7F3 epitope of P6 gene therefore 7F3 monoclonal antibody detects NTHi only
	Immuno-blot	109 (63 HT, 46 SP)	88 (44 ME, 44 HT)	McCrea et al., 2008; Sandstedt et al., 2008	P6 detected ~97% of NTHi strains and ~12% of *H. haemolyticus* strains and therefore is not completely discriminatory
	PCR	1 (LS)	12 (LS)	Abdeldaim et al., 2009	*ompP6* PCR amplified both *H. influenzae* and *H. haemolyticus*
	PCR	12 (oropharyngeal)	151 (50 oropharyngeal, 50 nasopharyngeal, 51 MEE)	Chang et al., 2010	P6 is not completely conserved in NTHi, and not all NTHi can be reliably distinguished from *H. haemolyticus* with *ompP6* sequence analysis and 7F3 reactivity
	PCR HRM	28 (27 NP, 1 ref)	27 (21 NP, 6 ref)	Binks et al., 2012	*ompP6* PCR and PCR HRM were both 94% sensitive, and 63% and 67% specific respectively for study-defined NTHi
			11 equivocal (11 NP)		
*rnbP*	PCR	1 (LS)	12 (LS)	Abdeldaim et al., 2009	*rnpB* PCR amplified both *H. influenzae* and *H. haemolyticus*
*bexA*	PCR	1 (LS)	12 (LS)	Abdeldaim et al., 2009	*bexA* PCR did not amplify *H. haemolyticus* but also failed to amplify 9 *H. influenzae* isolates
LOS genes *(licA*, *lic2A*, and *lgtC)*	PCR, DNA hybridization, Southern and Western blot	109 (63 HT, 46 SP)	88 (44 MEE, 44 HT)	McCrea et al., 2008, 2010b	*licA*, *lic2A*, and *lgtC* genes were more prevalent in *H. influenzae* isolates, but variable presence in *H. haemolyticus* means they are unsuitable targets for discrimination. Other *lic* genes found in *H. influenzae* and *H. haemolyticus. lic1* locus not part of the conserved core genome
	PCR	28 (27 NP, 1 ref)	27 (21 NP, 6 ref)	Binks et al., 2012	*lgtC* PCR did not amplify all NTHi strains and was therefore 80.6% sensitive and 94.8% specific for study-defined NTHi
			11 equivocal (11 NP)		
*iga*	Micro-array, dot blot	50 (HT, SP, micro-array)	50 (HT, SP and ME, micro-array)	McCrea et al., 2008; Sandstedt et al., 2008	The *iga* variable region probe used in dot blot hybridization was 100% accurate for categorizing strains that had been previously defined by their *adk, pgi, recA, infB* and *16SrDNA* sequences
		109 (HT, SP, dot blot)	88 (HT, SP and ME, dot blot)		
	IgA1 protease activity, DNA seq	7 (1 ref, 5 SP, 1 dental plaque)	8 (1 PS, 1 SP, 1 UN, 1 CSF, 3 blood, 1 LS)	Norskov-Lauritsen et al., 2009	*iga* was found to have limited discriminatory value for NTHi and *H. haemolyticus* differentiation
	PCR	28 (27 NP, 1 ref)	27 (21 NP, 6 ref)	Binks et al., 2012	The *iga* PCR was 88.9% sensitive and 91.7% specific for study-defined NTHi
			11 equivocal (11 NP)		
House-keeping genes	MLST	7 (1 LS, 6 variants)	90 (1 LS, 1 COPD, 88 MLST database)	Murphy et al., 2007	Variant strains did not encode *fucK*, but clustered with *H. haemolyticus*, and were distinct from *H. influenzae*
	MLST	7 (1 ref, 5 SP, 1 dental plaque)	8 (1 PS, 1 SP, 1 unknown origin, 1 CSF, 3 blood, 1 LS)	Norskov-Lauritsen et al., 2009	*fucK* was absent in 32 study strains (consisting of 7 *H. haemolyticus*, 8 NTHi, 1 *H. aeqyptius*, 2 variant haemophili, 4 Genospecies biotype IV and 20 *H. intermedius*) and removed from multi-locus sequence analysis. Strains of the *H. influenzae* cluster are more similar than variant strains
	DNA seq, PCR, Neighbor joining tree analysis, MLST	1 (LS)	627 (MLST database, 7 ref, 1 LS, 31 variants)	Ridderberg et al., 2010	*H. influenzae* may be untypable by MLST due to complete deletion of the fucose operon in some isolates
*hpd*	RTPCR	17 (1 invasive sequencing), 16 carriage isolates (sens/spec calcs)	247 (6 capsulate strains, 4 invasive NTHi (sequencing). 208 invasive, 29 carriage isolates (sens/spec calcs)	Wang et al., 2011	*hpd*#3 RTPCR was designed from *H. influenzae* specific regions of *hpd* and was found to be sensitive and specific for *H. influenzae* detection in CSF samples
	RTPCR	44 (44 PS carriage)	78 (78 PS carriage)	Theodore et al., 2012	9 of 78 *H. influenzae* isolates were not positive for *hpd*#3 RTPCR possible because of deletions of sequence variation
	RTPCR	28 (27 NP, 1 ref)	27 (21 NP, 6 ref)	Binks et al., 2012	*hpd*#3 RTPCR outperformed other *H. influenzae* assays with 88.9% sensitivity and 91.7% specificity
			11 equivocal (11 NP)		
	HRM-PCR	32 (1 ref, 31 NP) sequencing, 54 (NP, BAL, throat) HRM	138 (NP, BAL, throat) for HRM	Pickering et al., 2014a	*hpd* HRM PCR was 96% sensitive and 92% specific compared to the *hpd*#3 assay however *hpd* was not present in 11% of strains
*omp p2*	PCR	28 (27 NP, 1 ref)	27 (21 NP, 6 ref)	Binks et al., 2012	*omp* P2 PCR was 81% sensitive and 92% specific for study-defined NTHi
			11 equivocal (11 NP)		
*recA*	DNA seq	28 (27 NP, 1 ref)	27 (21 NP, 6 ref)	Binks et al., 2012	*recA* genes from NTHi and *H. haemolyticus* isolates did not cluster separately in all cases. Equivocal isolates and isolates related to *H. parainfluenzae* were intermingled between the predominant NTHi and *H. haemolyticus* clusters
			11 equivocal (11 NP)		
	DNA seq of concatenated *16SrDNA* and *recA*	20 (PS-asymptomatic)	52 (5 PS-asymptomatic, 47 PS from patients with URTI)	Zhu et al., 2013	*16SrDNA*-*recA* phylogeny is used to compare new differentiation methods of NTHi/*H. haemolyticus* such as MALDI-TOF (which is much faster)
*fucK*	Hybridization blots	1 (LS)	480 (241 RS, 161 MEE, 68 CS, 10 from other sites-2 blood)	Norskov-Lauritsen, 2009	*fucK* outperformed *hap* and *sodC* targets for *H. influenzae* identification
	Sequencing, PCR, Neighbor-joining tree analysis	1 (LS)	627 (MLST database, 7 ref, 1 LS, 31 variants)	Ridderberg et al., 2010	Occasional isolates of *H. influenzae* lack the entire fucose operon including *fucK*
	PCR	44 (44 PS carriage)	78 (78 PS carriage)	Theodore et al., 2012	22/78 *H. influenzae* isolates were not positive for *fucK* PCR and one *H. haemolyticus* was positive
	PCR	28 (27 NP, 1 ref)	27 (21 NP, 6 ref)	Binks et al., 2012	*fucK* PCR was 75% sensitive and 75% specific for study-defined NTHi
			11 equivocal (11 NP)		
	qPCR	1 (LS)	12 (LS)	Abdeldaim et al., 2013	Specifically detects *H. influenzae* and not the *H. haemolyticus* strain tested
*hap*	Hybridization blots	1 (LS)	480 (241 RS, 161 MEE, 68 CS, 10 from other sites-2 blood)	Norskov-Lauritsen, 2009	*hap* probe did not perform well because it failed to hybridize some *H. influenzae* and hybridized to several isolates excluded from *H. influenzae*
Antibiotic resistance genes	RTPCR	50 (URT)	50 (URT)	Witherden et al., 2013	Phenotypic and genotypic β-lactam resistance is present in NTHi and *H. haemolyticus* therefore PCRs based on resistance genes are not discriminatory for either species
Mass-spectrometry	MALDI-TOF MS (Bruker)	20 (PS-asymptomatic)	52 (5 PS-asymptomatic, 47 PS-URTi)	Zhu et al., 2013	Extending the original reference database provided with the Bruker biotype software to include more strains enabled 100% detection of NTHi and *H. haemolyticus* isolates to species level
	MALDI-TOF-MS (Bruker/Shimadzu)	7 (1 ref, 2 SP, 1 BAL, 1 PS, 1 U, 1 GF)	50 (8 SP, 9 NP, 8 TS, 4 BAL, 4 PS, 6 ES, 5 NS, 1 BS, 2 EARS, 1 MSS, 1 ref)	Frickmann et al., 2013	The Bruker MALDI-TOF device outperformed the Shimadzu, but MALDI-TOF was not completely discriminatory for NTHi and *H. haemolyticus*

a ,unknown site of isolation;

b , data not shown;

c , unclear whether typeable or non-typeable H. influenzae strains were assessed.

BAL, bronchoalveolar lavage; BLNAR, β-lactam negative ampicillin resistant; BS, bronchial secretion; CS, conjunctival swab; CSF, cerebrospinal fluid; EARS, ear swab; ES, eye swab; FISH, fluorescent in situ hybridization; GS, gastric fluid; HT, healthy throat; HRM, high resolution melt; IEF, isoelectric field; LS, laboratory strain; MALDI-TOF MS, matrix assisted laser desorption ionization time of flight mass spectrometry; MEE, middle ear effusion; ME, middle ear; MLSA, multilocus sequence analysis; MLST, multilocus sequence typing; MSS, Maxillary sinus swab; NS, nasal secretion; NP, nasopharyngeal; PCR, polymerase chain reaction; PS, pharyngeal swab; qPCR, quantitative PCR; ref, reference strain; RT, respiratory tract; sens/spec calcs, sensitivity and specificity calculations; SDS PAGE, sodium dodecyl sulfate polyacrylamide gel electrophoresis; seq, sequencing; SP, sputum; TS, tracheal secretion; U, urine; URT, upper respiratory tract; URTi, URT infection; UN, unknown origin.

## Genetic targets investigated for discrimination of NTHi from *H. haemolyticus*

The original discriminatory method used to distinguish NTHi from *H. haemolyticus* was a combination of *16SrDNA* PCR, a monoclonal antibody targeting an epitope of the outer membrane protein (OMP) P6 of NTHi known as 7F3, and multilocus sequence analysis (MLSA) (Murphy et al., 2007). *16SrDNA* PCR permitted easy identification of NTHi and *H. haemolyticus*, but only for 90% of strains. Recognition of the limitation of *16SrDNA* PCR as a discriminatory tool for NTHi and *H. haemolyticus* is now widely accepted (Norskov-Lauritsen, 2011; Binks et al., 2012). In 2011, Norksov-Lauritsen further investigated the apparent low resolution of classification schemes based on *16SrDNA* (Norskov-Lauritsen, 2011). *16SrDNA* genes are historically recognized as being universally distributed and therefore appropriate targets for assessing lineages. However, further investigation into the NTHi/*H. haemolyticus* species border found high numbers of polymorphic nucleotide positions due to intragenomic *16SrDNA* gene heterogeneity in isolates that were not NTHi. The increased level of *16SrDNA* gene polymorphism in commensal taxa (not including pathogenic *H. influenzae*) could not be explained but did provide a reason for the difficulties of *Haemophilus* speciation using *16SrDNA* gene-based classification. The 7F3 monoclonal antibody was found to be NTHi-specific and had the best differentiation capability, however its limited availability meant that widespread use of this method was unfeasible. Moreover, due to the cost and time, immunoblotting is not ideal for species identification in clinical diagnostic settings and subsequent studies have found that the 7F3 antibody does not identify all NTHi strains (McCrea et al., 2008). Multilocus sequence typing (MLST) is a standardized sequence-based profiling system that has been used to investigate NTHi diversity (Kaur et al., 2011; Schumacher et al., 2012; Puig et al., 2013) but, as discussed later, is not suitable for discrimination of NTHi from *H. haemolyticus*. MLSA is an extension of MLST that involves application of mathematical algorithms to assemble consensus trees (Tateno et al., 1994). In the Murphy study, MLSA identified that *H. haemolyticus* strains clustered separately to NTHi strains. Although MLSA is useful for understanding species boundaries, it requires a high level of technical expertise and is time consuming and therefore not ideal for routine diagnostics.

Another molecular target with the potential ability to completely differentiate NTHi from *H. haemolyticus* was simultaneously described by Fung et al. (2006). The *sodC* gene, which encodes the copper- and zinc-containing superoxide dismutase CuZnSOD, was found to be present in 20 *H. haemolyticus* isolates and absent in 20 NTHi isolates. Initial PCR results were confirmed by Southern and Western blotting. However, subsequent application of the *sodC* PCR to a larger collection of isolates in 2010 (110 *H. haemolyticus* and 169 NTHi) revealed that 9% of NTHi also possessed the *sodC* gene (McCrea et al., 2010a), demonstrating that the *sodC* gene was not a suitable target for complete discrimination of NTHi from *H. haemolyticus*.

In 2008, McCrea et al. thoroughly investigated the relationship of NTHi to hemolytic and non-hemolytic *H. haemolyticus* strains (McCrea et al., 2008). Taxonomic traits, MLSA and the presence of NTHi virulence-associated genes encoding lipooligosaccharide (*licA, lic2A, lgtC*), and IgA protease were compared. Eighty-eight capsulated and non-typeable *H. influenzae* (breakdown not given), and 109 culture-defined *H. haemolyticus* isolates were examined. The 109 *H. haemolyticus* isolates were not bound by *iga* hybridization probes, and this was the only target that differentiated all *H. influenzae* and *H. haemolyticus* isolates in the study. Whilst taxonomic traits such as H_2_S and indole production, urease and ornithine decarboxylase activity and hemolysis (Kilian, 1976b,a) were found to correlate with species identification, no trait completely differentiated NTHi from *H. haemolyticus* (McCrea et al., 2008). A main finding was that although hemolytic and non-hemolytic *H. haemolyticus* strains did not cluster as two separate subspecies, some NTHi genes (*licA*) and traits (urease activity) were more common in hemolytic strains compared with non-hemolytic strains. The authors remarked that no rapid, clinically useful marker was available to differentiate NTHi and *H. haemolyticus*, however X and V factor testing was sufficient for distinguishing *H. influenzae* from other haemophili that infect normally sterile sites and cause serious disease. The authors proposed that precise taxonomic division of these species is elusive, particularly with the high potential for genetic recombination between NTHi and *H. haemolyticus* that has since been demonstrated *in vitro* (Sondergaard et al., 2014). At this stage *H. haemolyticus* had not been associated with disease: there were two rare cases of *H. haemolyticus* causing endocarditis in 1923 (De Santo and White, 1933) and 1933 (Miller and Branch, 1923). The recent retrospective molecular analysis of NTHi culture-defined isolates has revealed additional cases in which *H. haemolyticus* was the apparent cause of bacteremia and septic arthritis (Anderson et al., 2012; Morton et al., 2012). Although *H. haemolyticus* infection is still rare, these cases reiterate the need for specific identification tools.

In 2008, Sandstedt et al. compared the generation of minimum evolution trees from concatenated sequences of 5 housekeeping genes (*adk, pgi, recA, infB*, and *16SrDNA*), which had previously been shown to be the best method for distinguishing NTHi from *H. haemolyticus* (Norskov-Lauritsen et al., 2005; McCrea et al., 2008), with rapid and more cost-effective methods (Sandstedt et al., 2008). The three methods evaluated were DNA hybridization-based microarrays (targeting conserved and variable *iga* regions), genomic dot blot hybridization (also targeting conserved and variable *iga* regions), and dot blot immunoassays for OMP P6 with monoclonal antibody 7F3. Genomic dot blots targeting the *iga* variable region correlated most closely with the minimum evolution trees, whereas microarray detection of the variable *iga* region was favored for being high-throughput. Methods utilizing the conserved portion of the *iga* gene did not discriminate NTHi from *H. haemolyticus*. The authors recognized that the adoption of phylogenetic or molecular methods for NTHi/*H. haemolyticus* differentiation is dependent on the number of strains being analyzed and the purpose of doing so, which varies from laboratory to laboratory.

In 2009, the *sodC*, *fucK* (encodes fuculokinase), and *hap* (haemophilus adhesion protein) genes were investigated for their combined suitability to selectively identify NTHi (Norskov-Lauritsen, 2009). *H. influenzae* isolates (typeable and non-typeable) were expected to be *sodC*−, *fucK*+, and *hap*+. The *fucK* PCR gave the best discrimination between the 480 isolates investigated. It was suggested that phenotypic *H. influenzae* isolates lacking *fucK* were not *H. influenzae* and development of a *fucK*- based molecular discriminatory tool was proposed. Soon after this publication, the same group published a more detailed investigation into the delineation of *H. influenzae* by phenotype, multilocus sequence phylogeny and detection of marker genes (*16SrDNA, hap, fucK, sodC*, and virulence-associated genes *hia, hmw1A, hmwC, hif, iga, lic2B*) (Norskov-Lauritsen et al., 2009). In this study, the species borders for *H. influenzae* with (1) *H. haemolyticus*, (2) cryptic genospecies biotype IV, and (3) the then un-validated species “*H. intermedius”* were interrogated with MLSA for 6 of the 7 MLST housekeeping genes: *adk*, *atpG*, *frdB*, *mdh*, *pgi*, and *recA* (*fucK* was excluded from the MLST due to its absence in 42 strains). Individually, *16SrDNA*, *hap, fucK*, and *sodC* genes correlated with the concatenated multilocus sequence phylogeny, but *iga* was found to have limited discriminatory value for NTHi and *H. haemolyticus* differentiation. This contrasted with previous studies detailing the discriminatory power of *iga* for *H. influenzae* detection (McCrea et al., 2008; Sandstedt et al., 2008). The virulence associated markers *hia, hmw1a, hmwC*, and *hif* were variably expressed in *H. influenzae* and therefore not discriminatory. Multilocus sequence phylogeny of *H. haemolyticus* strains produced separate lineages that also included *H. intermedius* and the cryptic genospecies biotype IV. This finding emphasized the difficulty of defining taxonomic boundaries within *Haemophili*. The authors observed that sequence analysis did not align with taxonomy, and suggested that different strains of *H. haemolyticus* may not share a common ancestor.

By 2010, it became apparent that not all strains of *H. influenzae* encode the *fucK* house-keeping gene, with some strains missing the entire fucose operon (Ridderberg et al., 2010). Therefore, the standardized MLST assay for *H. influenzae* is not suitable for all strains. In the same year, another study reported on variations in the OMP P6 (*omp P6*) gene of NTHi that obscures NTHi and *H. haemolyticus* differentiation (Chang et al., 2010). At this stage, none of the previously characterized targets remained attractive candidates for accurate identification of *H. influenzae* or *H. haemolyticus*.

Real-time (RT) PCR assays targeting *16SrDNA* (Abdeldaim et al., 2009), *omp P6* (Nelson et al., 1991; Abdeldaim et al., 2009), *bexA* (Corless et al., 2001), *rnpB* (Abdeldaim et al., 2009), and *fucK* (Abdeldaim et al., 2013) genes were developed for diagnostic detection of *H. influenzae*, however each was limited by poor specificity and/or sensitivity. For example, the *rnpB* RTPCR amplifies both NTHi and *H. haemolyticus* (Abdeldaim et al., 2009), whereas the *bexA* PCR does not amplify *H. haemolyticus*, but also failed to amplify all *H. influenzae* strains (Corless et al., 2001). In 2011, a quantitative RTPCR (*hpd#*3) based on the protein D gene (*hpd*) was developed that was sensitive and appeared to be specific for *H. influenzae* identification (Wang et al., 2011). Sixteen *H. haemolyticus* isolates were tested with the *hpd#3* RTPCR and none were positive. A major advantage of the *hpd#*3 RTPCR was that it could be used directly on clinical samples, reducing cost and preparation time for *H. influenzae* identification. In 2012, the *hpd#*3 RTPCR assay was further investigated in a collection of 60 culture-defined NTHi from the nasopharynx of children with and without recurrent acute otitis media (Binks et al., 2012). *16SrDNA* PCR had previously identified that only 37% (22/60) of the isolates were true NTHi, 27 were *H. haemolyticus* and the remaining 18% (11/60) could neither be defined as NTHi nor *H. haemolyticus* and were termed equivocal (Kirkham et al., 2010). Sequencing and concatenation of *16SrDNA* and *recA* genes in this collection of isolates provided insight into the previously ambiguous equivocal isolates. Whilst *16SrDNA* PCR-defined *H. haemolyticus* and NTHi strains were clearly separated from one another on the phylogenetic tree, the equivocal strains sat in the middle and were considered to either be divergent *H. haemolyticus* strains becoming NTHi or *vice versa*. An evolutionary continuum between the two species was suggested. This collection of isolates was considered to be ideal to test the limitations of existing discriminatory assays in their ability to identify the NTHi and *H. haemolyticus*. Seven of the most promising PCR targets (*hpd*, *omp P2, omp P6, lgtC, 16SrDNA, fucK*, and *iga*) were assessed (Binks et al., 2012). The study conceded that NTHi and *H. haemolyticus* could not be completely differentiated with any single gene target, however the *hpd#3* RTPCR was superior for differentiating closely related strains. A subsequent study suggested conducting 3 tests: *hpd#3* RTPCR, *fucK* PCR and then *16SrDNA* sequencing for NTHi and *H. haemolyticus* differentiation (Theodore et al., 2012). However, the identification of strains lacking *fucK* remains an issue for its broad application, and conducting 2 PCRs followed by sequencing increases the cost and time for identification. Such lengthy and expensive tests are not ideal for clinical diagnostics or large-scale surveillance studies.

Recently, we developed a HRM (high resolution melt)-PCR to further investigate the potential use of the *hpd* gene to detect and differentiate NTHi and *H. haemolyticus* (Pickering et al., 2014a). The advantage of the PCR-HRM is low cost, speed and that only one reaction is required for differentiation of the two species. However, application of this assay to 180 clinical isolates revealed that even *hpd*, a host colonization-associated gene (Johnson et al., 2011) that was previously reported to be highly conserved (Song et al., 1995), was not present in 11% (19/180) of the isolates tested. Absence or variability of the *hpd* gene in NTHi has since been confirmed (Zhu et al., 2013; Smith-Vaughan et al., 2014) and suggests that the *hpd* gene is less conserved than originally thought. The *hpd* HRM-PCR is limited like all other single-target tools tested to date. Summarizing all *H. influenzae*/*H. haemolyticus* differentiation studies, it appears that single gene target approaches for discrimination are not ideal and that rapid tests incorporating multiple targets are required.

## Proteomic and whole genome approaches to discrimination of NTHi and *H. haemolyticus*

Matrix-assisted laser desorption/ionization time of flight (MALDI-TOF) analysis is revolutionizing the diagnostic laboratory. MALDI-TOF compares the spectral profiles of bacterial colonies to a database of species with known spectral profiles. The first assessment of the utility of MALDI-TOF to differentiate *H. influenzae* from *H. haemolyticus* reported 100% differentiation of 52 NTHi and 20 *H. haemolyticus* strains with the adaptation of a new reference database on the Bruker MALDI-TOF platform (Zhu et al., 2013). A second study compared two MALDI-TOF platforms (Shimadzu and Bruker) with fluorescent *in situ* hybridization (FISH) to identify 50 *H. influenzae*, 25 *H. parainfluenzae*, 7 *H. haemolyticus*, and 2 *H. parahaemolyticus* isolates (Frickmann et al., 2013). FISH failed to identify strains from all *Haemophilus* species tested including a high proportion (14%) of *H. influenzae* isolates. Neither MALDI-TOF platform correctly identified any *H. haemolyticus* isolates but addition of an *H. haemolyticus* reference spectrum to the Bruker database resulted in identification of all seven *H. haemolyticus* isolates. This demonstrates that the discriminatory power of MALDI-TOF is highly dependent on the comprehensiveness of species databases, which varies between laboratories. Improvement and global standardization of reference databases for *H. influenzae* and *H. haemolyticus* may permit the use of MALDI-TOF for high-throughput speciation of *H. influenzae* and *H. haemolyticus* in diagnostic laboratories in the near future.

An alternative approach to identifying and developing tools for multiple discriminatory targets of *H. influenzae* and *H. haemolyticus* is the use of whole genome sequencing and comparative genomics. There are several large-scale *Haemophilus* whole genome sequencing projects underway that will assist in development of such methods. Recently, comparison of 97 NTHi genomes revealed an NTHi population structure of 6 distinct clades. This high-resolution study is the first to identify a clonal-based evolution of NTHi (De Chiara et al., 2014). *H. haemolyticus* strains were not included in this study.

In summary, although the need for molecular identification is acknowledged, no single target or current methodology has been identified that can accurately identify all *H. influenzae* or *H. haemolyticus* strains. This is further complicated by the genetic relatedness of these species and the demonstration that inter-species horizontal gene transfer occurs. When new discriminatory tests are developed they must be validated on a large and diverse collection of strains. Future large-scale comparative genomic studies that compare *H. influenzae* core and accessory genes with *H. haemolyticus* have the potential to reveal new discriminatory targets and provide greater definition of species borders. This in turn will improve the accuracy of *H. influenzae* and *H. haemolyticus* identification for improved disease diagnosis and surveillance.

## Author contributions

Janessa Pickering prepared the manuscript, Peter C. Richmond and Lea-Ann S. Kirkham critically reviewed the manuscript. The authors do not have any competing interests.

### Conflict of interest statement

The authors declare that the research was conducted in the absence of any commercial or financial relationships that could be construed as a potential conflict of interest.
